# Associated factors and rise of burnout in Mexican teachers: the impact of school principals, age, and years of teaching experience

**DOI:** 10.3389/fpsyg.2025.1378122

**Published:** 2026-01-29

**Authors:** Angelica Janeth Cortez Soto, Yolanda Heredia Escorza

**Affiliations:** Tecnologico de Monterrey, Monterrey, Mexico

**Keywords:** burnout syndrome, stress, teacher burnout, school teachers, working conditions, burnout factors

## Abstract

Burnout is a major occupational issue, particularly among teachers, who face high emotional and physical demands. Despite extensive research, few studies have compared personal and job-related predictors simultaneously. This study examined these factors among preschool and elementary teachers in Mexico, and assessed overall burnout levels. A cross-sectional quantitative study was conducted with 637 teachers using the Spanish Burnout Inventory. Data were analyzed with classification tree analysis and non-parametric tests (Mann–Whitney *U*, Kruskal–Wallis). Results show that principal support is the strongest protective factor, while excessive demands, fewer years of experience, younger age, grade level taught, and urban location increase burnout risk. Overall levels were moderate across dimensions. These findings highlight the need for interventions, especially in urban schools and early grades. Leadership training for principals and workshops to strengthen teachers’ emotional regulation and relationship management are recommended to promote teacher well-being.

## Introduction

Burnout has emerged as a central concern in occupational health, especially in professions characterized by sustained emotional demands. The International Labour Organization (2015) emphasized that prolonged exposure to stressors such as excessive workloads, role ambiguity, organizational change, lack of job satisfaction, poor work–life balance, inadequate workplace relationships, insufficient support, and even violence or harassment can result in burnout. Reflecting its relevance, the International Classification of Diseases, 11th Revision (ICD-11), officially recognizes burnout as an occupational phenomenon described by the World Health Organization (2019) as energy depletion, increased cynicism, and reduced professional efficacy.

The conceptualization of burnout has been shaped through theoretical models and measurement tools. Maslach et al. (2001) defined it as a psychological response to persistent emotional and interpersonal stressors at work and developed the Maslach Burnout Inventory (MBI), which evaluates emotional exhaustion, depersonalization, and diminished personal accomplishment. Although the MBI was the first standardized tool, subsequent critiques and diverse perspectives have led to alternative instruments and revealed the lack of consensus around a universal definition (Grow et al., 2019; Hewitt et al., 2020).

Building on this diversity, Gil-Monte (2005) introduced the Spanish Burnout Inventory (SBI), which conceptualizes burnout as a result of chronic stress in interpersonal work relations and includes reduced enthusiasm, psychological exhaustion, indolence, and guilt. The model also distinguishes between two profiles: Profile 1, marked by moderate stress and diminished enthusiasm but limited guilt, and Profile 2, associated with severe stress, guilt, and even absenteeism (Gil-Monte, 2012). This framework underscores how burnout may vary in intensity and progression among professionals.

The prevalence of burnout is particularly high in professions centered on care and service provision, including teaching, medicine, psychology, nursing, and social work (Bouza et al., 2020). In Mexico, official reports describe manifestations such as inefficiency, fatigue, and detachment, especially among individuals in their 30s and 40s (Secretaría de Salud, 2015). The urgency of this issue became evident during the COVID-19 pandemic, when the Mexico City Congress highlighted the severe exhaustion of educators and called for institutional responses to prevent and address burnout (Congreso Ciudad de México, 2021).

Teachers work under conditions of continuous emotional and physical strain, which increase their vulnerability to burnout. Conflicts with colleagues, challenging students, pressure from families, large class sizes, limited resources, low salaries, and administrative overload are among the most common stressors (Madigan et al., 2023; Vega-Feijoó et al., 2020). Alongside these factors, research has examined personal and professional characteristics as potential determinants of burnout and teacher performance.

Age and years of professional experience have often been studied in relation to teaching performance, yet findings remain mixed. Some evidence suggests that experience strengthens skills, while other studies note that aging and growing job demands may offset these benefits. Thus, age and experience cannot fully account for performance differences and must be considered in interaction with contextual variables (Pranoto et al., 2021).

The gender imbalance in teaching is a global phenomenon, with a steady decline in male teachers reported across multiple contexts (McGrath and Van Bergen, 2017; OECD, 2016; UNESCO, 2011). Explanations include structural and cultural factors, such as low salaries and perceptions of teaching as a feminized profession, as well as dispositional traits, since female teachers tend to report stronger pedagogical and altruistic motivations while male teachers more frequently cite subject-related or pragmatic motives (Kammermeier et al., 2025; Pranoto et al., 2021). Taken together, these explanations illustrate how gender intersects with teacher performance and burnout risk.

Marital status and work–life balance are also significant in understanding burnout. Teachers often juggle family responsibilities and caregiving demands that affect their energy and opportunities to fulfill professional roles. Research highlights that role conflict and family obligations exacerbate stress and increase vulnerability to burnout.

Geographical and contextual disparities further shape teachers’ professional experiences. Urban schools generally benefit from stronger infrastructure and access to resources, whereas rural schools often face shortages of qualified staff and limited opportunities for development (Zhang et al., 2024). Nevertheless, rural contexts can also foster closer student–teacher relationships and place-based educational practices that mitigate burnout (Goodpaster et al., 2012; Hardré, 2013).

Marital status is a relevant dimension in understanding teacher burnout, particularly when analyzed from a gender perspective. Female teachers who are also mothers report greater difficulties in achieving work–life balance, as domestic and caregiving roles overlap with professional responsibilities (Barbarona-Gudelosao and Escote, 2025). Evidence from school contexts shows that this work–family conflict among female teachers is linked to higher stress, burnout risk, absenteeism, and intentions to leave, which undermines teacher retention (Atteh et al., 2020).

Teacher professional development and continuing training can strengthen educators as “high-level knowledge workers” who advance their professional expertise (Schleicher, 2012). Such opportunities are linked to greater wellbeing and improved classroom management when embedded in professional learning communities (Prilleltensky et al., 2016), and they are associated with stronger intentions to remain in the profession (George, 2015). Professional development also tends to bring changes in teaching practice, although its direct impact on student achievement is modest (Fischer et al., 2018).

Finally, school leadership plays a crucial role in shaping organizational climate and teacher well-being. Principals’ practices directly influence teacher stress and burnout, as supportive leadership and clear goal-setting contribute to healthier work environments (Agasisti et al., 2019; Dhuey and Smith, 2018). This underscores the importance of leadership as a protective factor in understanding teacher burnout.

Despite an expanding body of literature, few studies in Mexico have examined teacher burnout using validated instruments such as the SBI while integrating both personal and professional predictors. Therefore, this study seeks to compare the impact of factors such as age, gender, marital status, and teaching experience with professional variables including academic qualifications, school level, grade taught, location, and perceptions of leadership. Additionally, the study aims to measure overall burnout levels and their dimensions to assess the need for intervention.

## Method

The study employed a quantitative, descriptive, and cross-sectional design to examine the relationship between independent variables (including perceived support, guidance, and positive attitude of the school principal; perceived excessive demands of the principal; teachers’ educational background; age; years of experience; city of employment; school level; marital status; and grade taught) and the dependent variable, teacher burnout.

### Participants

In Mexico, basic education is divided into three stages according to students’ age: preschool, elementary school, and middle school. Preschool typically includes children aged 3–5 years, elementary school covers ages 6–11, and middle school encompasses ages 12–14 (Ríos Félix et al., 2020). This research focused on preschool and elementary school teachers because the characteristics that contribute to burnout are similar at these levels. At these stages, a single teacher is responsible for the entire class, unlike at the secondary level where teachers may teach one or several subjects to different groups and grades.

The study involved a sample of 637 active teachers, ranging in age from 21 to 72 years (*M* = 40.0). Of these, 542 (85.1%) were female and 95 (14.9%) were male. Teaching experience ranged from 0 to 53 years (*M* = 16.24). Regarding marital status, 407 (63.9%) were married, 156 (24.5%) were single, 62 (9.7%) were divorced, and 12 (1.9%) were widowed. In terms of professional qualifications, 483 (75.8%) held a bachelor’s degree, 144 (22.6%) had a master’s degree, 6 (0.9%) held a doctorate, and 4 (0.6%) had a high school diploma. Regarding work location, 533 (83.7%) of participants worked in urban areas, while 104 (16.3%) worked in rural areas. In terms of educational levels taught, 290 (45.5%) worked in preschool and 347 (54.5%) in elementary school. Participation was entirely voluntary, and a non-probabilistic convenience sampling method was employed.

### Instrument

The instrument employed was the Spanish Burnout Inventory (SBI) developed by Gil-Monte (2005). It consists of 20 items grouped into four dimensions:

- Enthusiasm toward the job—enjoyment and satisfaction derived from work; items are positively framed, with lower scores indicating higher levels of burnout.
- Psychological exhaustion—emotional and physical depletion resulting from daily interactions with individuals who present or cause problems in the workplace.
- Indolence—attitudes of indifference and cynicism toward students.
- Guilt—feelings of guilt, considered a core symptom in Gil-Monte’s theoretical model of burnout.

In addition to the SBI and personal data, participants answered two items assessing their perception of their workplace principal:

- “The principal of my workplace supports, guides, and exhibits a positive attitude.”
- “The principal of my workplace is excessively demanding.”

Responses to these items followed the same Likert-type scale as the SBI:

- 0 = Never.
- 1 = Rarely (occasionally throughout the year).
- 2 = Sometimes (occasionally throughout the month).
- 3 = Frequently (several times per week).
- 4 = Very frequently (every day).

### Procedure

The recruitment of participants was carried out in collaboration with educational authorities from the Ministry of Education of Nuevo León. An official memorandum describing the objectives and details of the study was disseminated to potential participants. Teachers received a link to a Google Forms survey that included both the research instrument and a section for demographic information. Prior to participation, each teacher was presented with an informed consent document, which emphasized the voluntary nature of their involvement and guaranteed the confidentiality of their responses. The study was conducted in accordance with ethical standards for research involving human participants, following the principles of the Declaration of Helsinki (World Medical Association, 2013) and the APA Ethics Code (American Psychological Association, 2017). Participants were informed of their right to withdraw at any time without penalty, and no identifying information was shared outside the research team.

### Data analysis

The study followed a quantitative, descriptive, and correlational design. Data were analyzed using the Statistical Package for the Social Sciences (SPSS). Firstly, descriptive statistics were calculated to examine the distribution of teachers across overall burnout levels and the four dimensions of the Spanish Burnout Inventory.

Next, Classification Trees, a data mining technique, were employed to explore the predictive relationship between independent variables (e.g., principal support, excessive demands, and personal demographics) and the dependent variable of teacher burnout (Pérez, 2011). To further analyze variables not included in the decision tree, group comparison tests were conducted. The Kolmogorov–Smirnov test, recommended for samples larger than 30, was used to assess normality, with results confirming a non-normal distribution (Hart, 2001).

Consequently, non-parametric tests were applied: the Mann–Whitney *U* test compared differences in burnout levels between two groups (e.g., city of employment, educational level taught), while the Kruskal–Wallis test was used to examine differences among three or more groups (e.g., marital status, educational qualifications, grade taught) (Flores-Ruiz et al., 2017). This analytic strategy provided a comprehensive overview of the factors associated with teacher burnout, generating insights to inform targeted interventions and support programs in educational settings.

## Results

**Figure 1:**
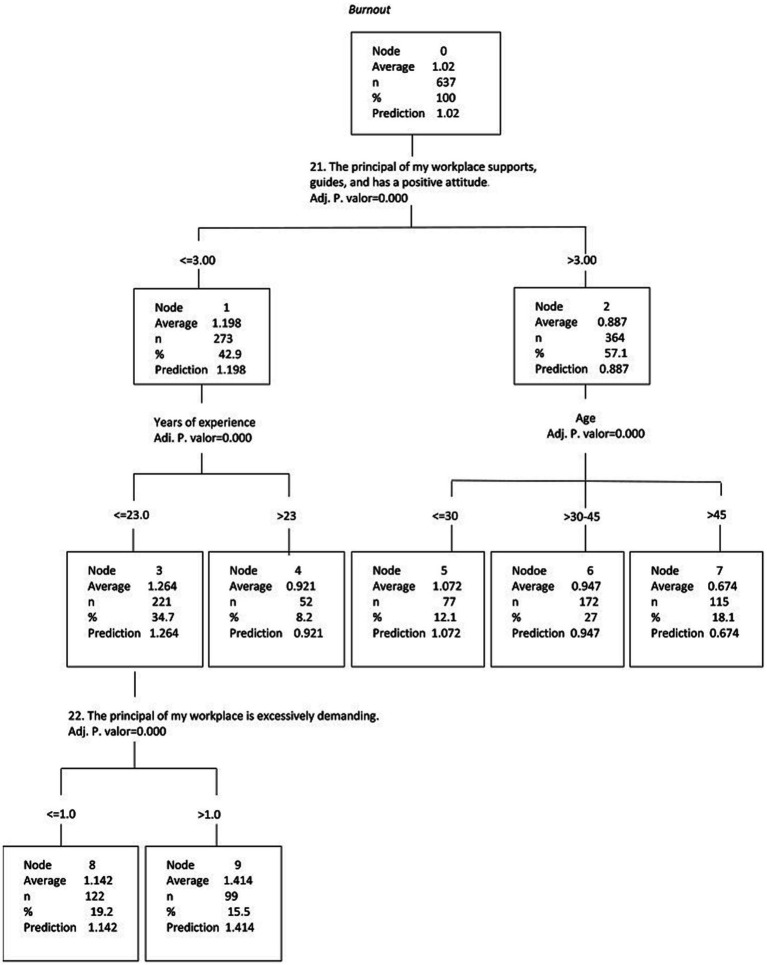
Decision tree with predictors of teacher burnout.

Figure 1 presents the classification tree generated using the CHAID method, which produced 10 nodes in total and 6 terminal nodes. Node 0 indicates the overall burnout mean (*M* = 1.02, SD = 0.516; *n* = 637). The strongest predictor of burnout was perceived principal support (*p* < 0.001). Teachers reporting very frequent support (score = 4) had lower burnout (*M* = 0.887; Node 2) than those reporting support less frequently (scores 0–3) (*M* = 1.198; Node 1).

- Node 1 then split by years of experience (*p* < 0.001). Teachers with > 23 years of experience reported lower burnout (*M* = 0.921; Node 4), while those with ≤ 23 years reported higher burnout (*M* = 1.264; Node 3). Within the latter group, those perceiving their principal as excessively demanding at least sometimes (scores ≥ 2) showed the highest burnout levels (*M* = 1.414; Node 9), whereas those perceiving such demands as never or rarely (scores 0–1) showed lower burnout (*M* = 1.142; Node 8).
- In the high-support branch (Node 2), burnout varied significantly by age (*p* < 0.001). Teachers ≤ 30 years old reported the highest burnout (*M* = 1.072; Node 5), those aged 30–45 reported moderate levels (*M* = 0.947; Node 6), and those > 45 years reported the lowest (*M* = 0.674; Node 7).

Table 1 summarizes the descriptive distribution of burnout for the full sample. Overall burnout concentrated in the medium range (42.2%), followed by low (22.8%) and high (18.2%), with critical cases at 5.2% and very low at 11.6%. By dimensions, the modal category was medium for Enthusiasm toward the job (40.82%), Psychological Exhaustion (38.62%), Indolence (39.87%), and Guilt (35.48%). Notably, Psychological Exhaustion and Indolence showed sizable upper-tier proportions (high/critical at 20.25%/18.21 and 27.16%/18.21%, respectively), whereas Guilt was comparatively lower in the upper tiers (8.95% high, 5.81% critical). These distributions indicate that while most teachers fall in the medium range across dimensions, exhaustion and indolence cluster more heavily toward higher severity than guilt.

**Table 1:** Distribution of teachers by level of burnout and burnout dimensions.

Level/burnout dimension	Enthusiasm toward the job	Psychological exhaustion	Indolence	Guilt	Burnout
Very low	43 teachers 6.75%	61 teachers 9.58%	25 teachers 3.92%	144 teachers 22.61%	73.89 teachers 11.6%
Low	102 teachers 16.01%	90 teachers 14.13%	69 teachers 10.83%	173 teachers 27.15%	145.25 teachers 22.8%
Medium	260 teachers 40.82%	246 teachers 38.62%	254 teachers 39.87%	226 teachers 35.48%	268.81 teachers 42.2%
High	232 teachers 36.42%	129 teachers 20.25%	173 teachers 27.16%	57 teachers 8.95%	115.93 teachers 18.2%
Critical		111 teachers 17.42%	116 teachers 18.21%	37 teachers 5.81%	33.12 teachers 5.2%

Summary of predictions:

- Perceived principal support (score = 4 = very frequent) emerged as the strongest predictor of teacher burnout.
- The highest burnout (*M* = 1.414) occurred among teachers with ≤ 23 years of experience, perceiving their principal as excessively demanding (sometimes, frequently, or very frequently), and reporting less than very frequent support.
- The lowest burnout (*M* = 0.674) occurred among teachers > 45 years old who reported very frequent principal support.

### Group comparisons

**Figure 2:**
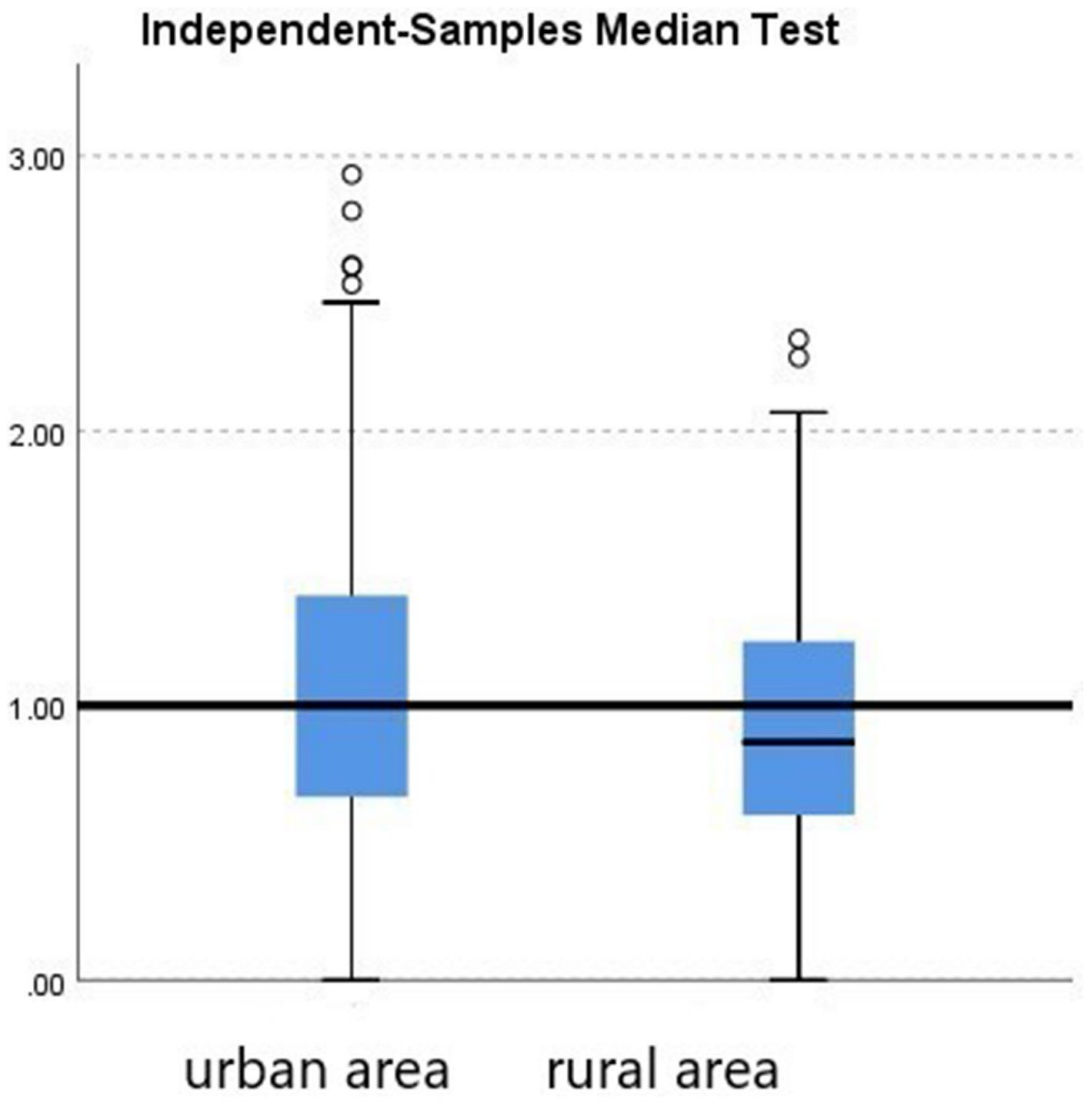
Boxplot of the Mann–Whitney *U* test for burnout level between an urban area and a rural area.

**Figure 3:**
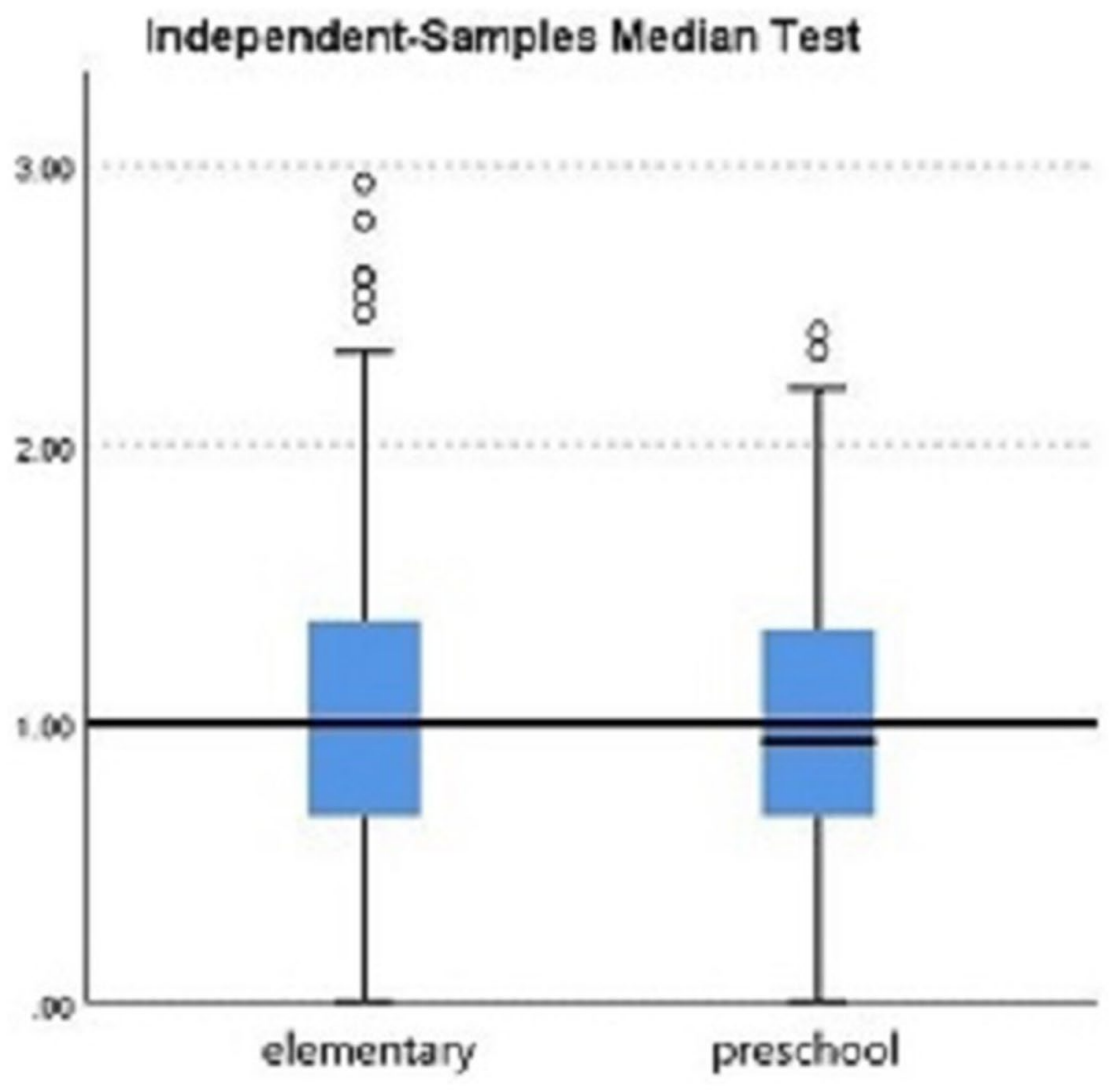
Box plot of the Mann–Whitney *U* test for burnout level between elementary and preschool levels.

**Figure 4:**
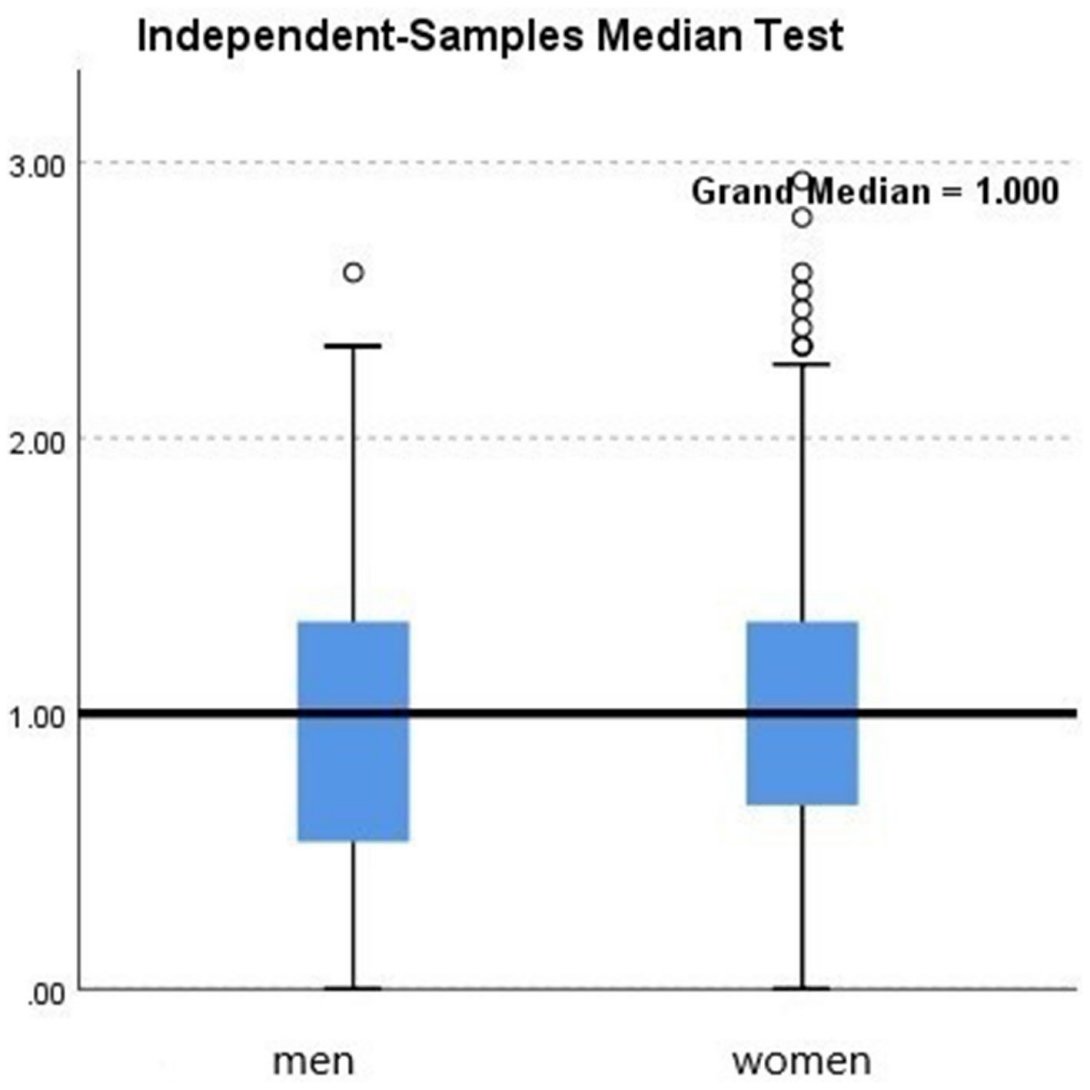
Box plot of the Mann–Whitney *U* test for the level of burnout according to gender.

**Figure 5:**
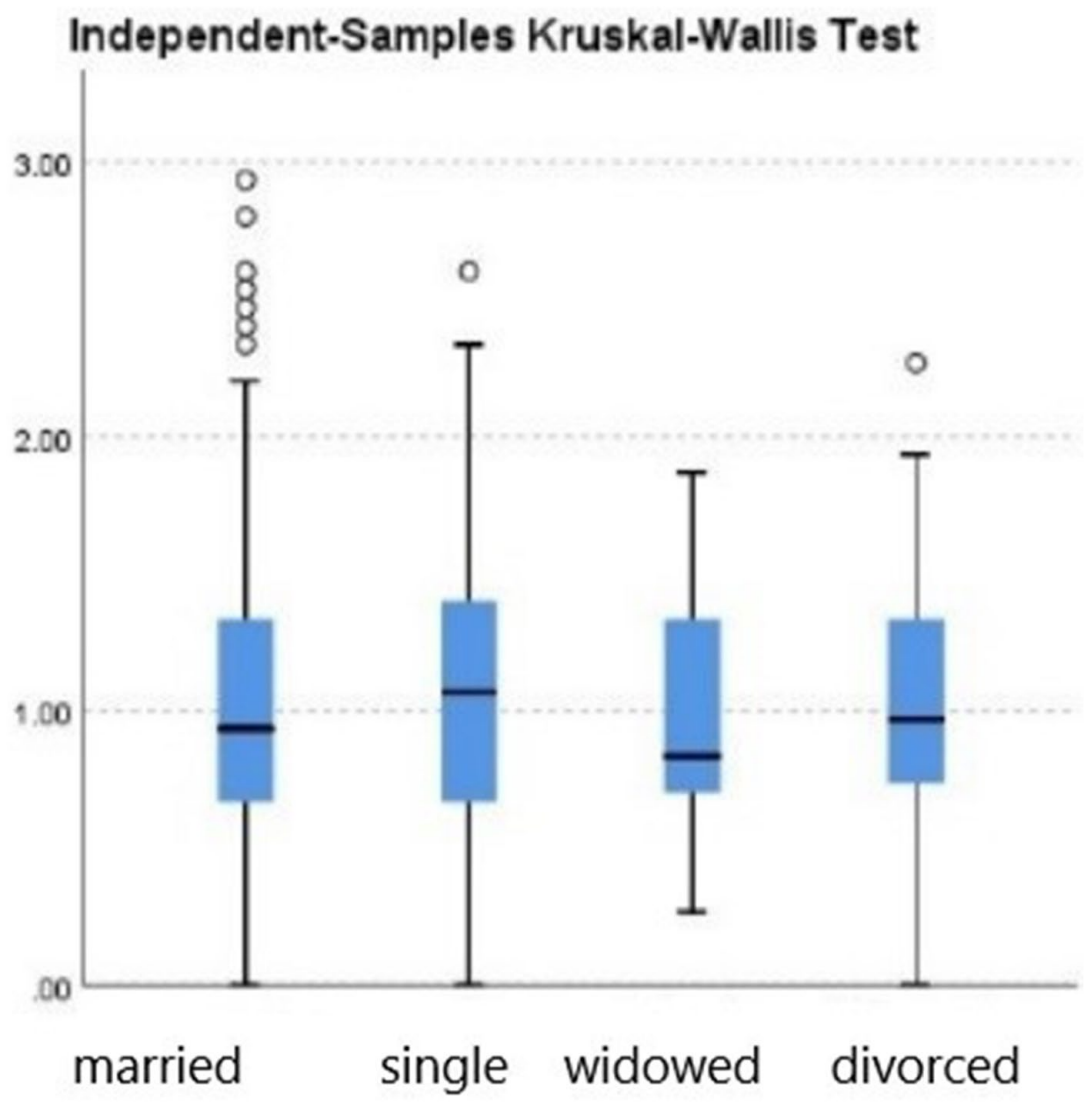
Box plot of the Kruskal–Wallis test for the teacher burnout levels among participants grouped by their marital status.

**Figure 6:**
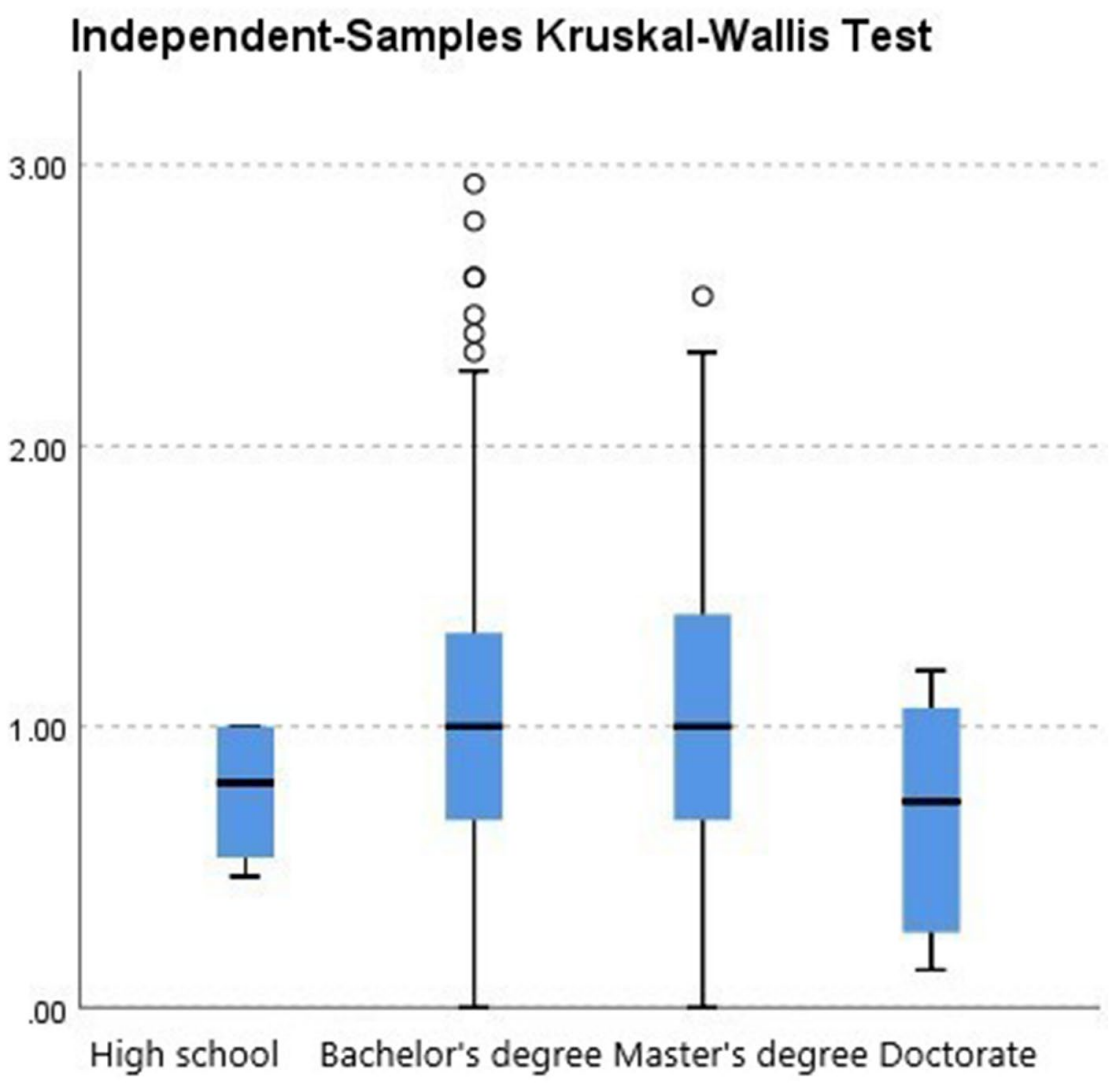
Box plot of the Kruskal–Wallis test for the teacher burnout levels among participants grouped by their highest level of education completed.

**Figure 7:**
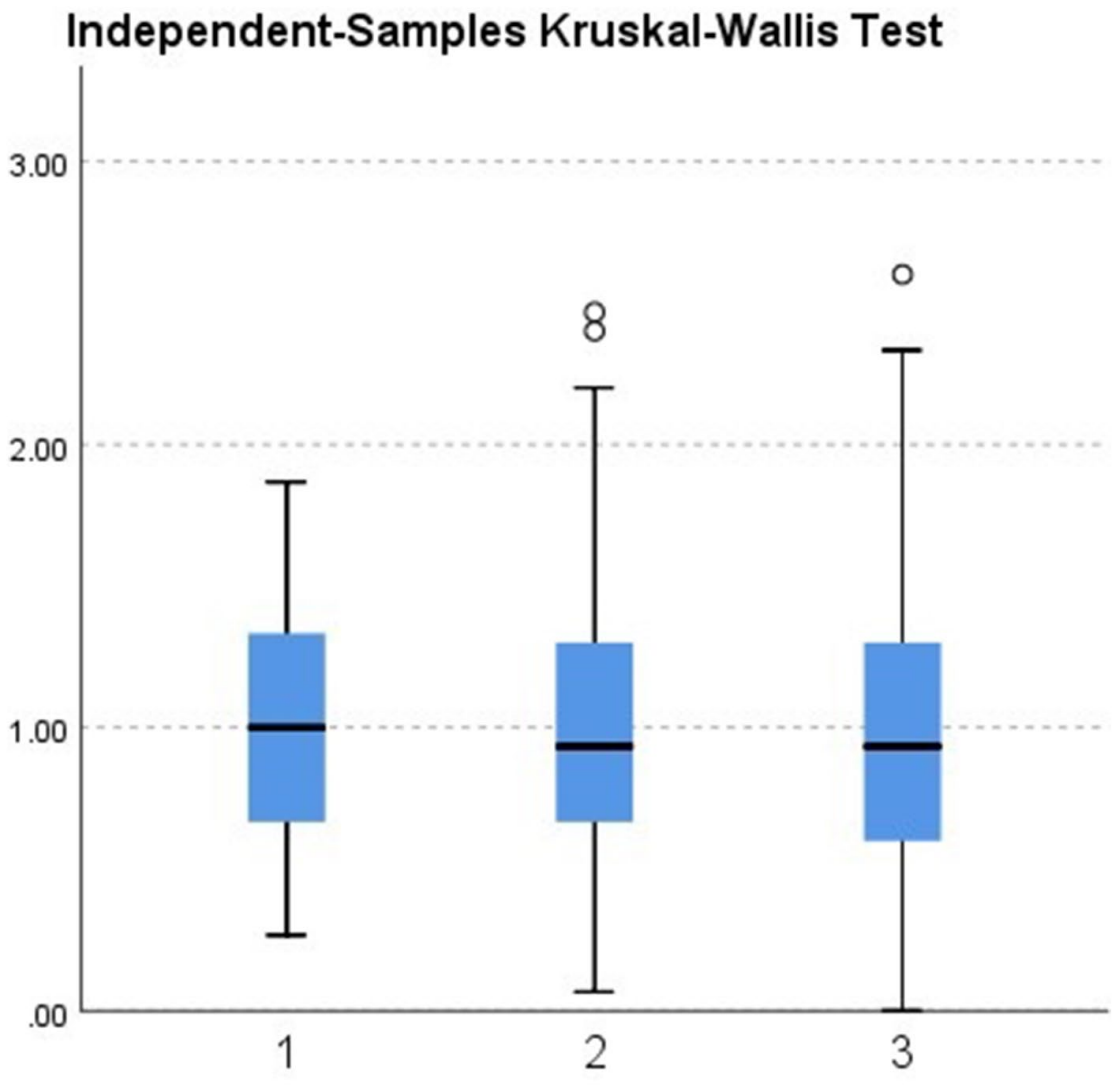
Box plot of the Kruskal–Wallis test for the burnout level of participants in the preschool level grouped according to the grade they teach.

**Figure 8:**
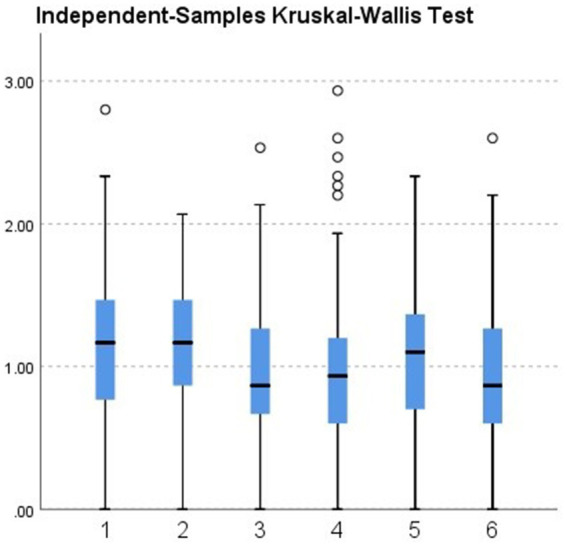
Boxplot of the Kruskal–Wallis test for the burnout level of participants in the elementary school level grouped according to the grade they teach.

Further group comparisons using non-parametric tests revealed the following:

- City (urban vs. rural). Teachers in urban areas showed significantly higher burnout levels than those in rural areas (*p* = 0.033; see Figure 2, Table 2).
- School level (preschool vs. elementary). Although not statistically significant (*p* = 0.448), elementary teachers had a slightly higher median burnout score compared with preschool teachers (see Figure 3, Table 3).
- Gender. Although not statistically significant (*p* = 0.182), women showed a slightly higher median burnout score compared with men (see Figure 4, Table 4).
- Marital status. Although differences were not statistically significant (*p* = 0.397), single teachers exhibited the highest median burnout, followed by divorced, married, and widowed teachers (see Figure 5, Table 5).
- Education level. Although not statistically significant (*p* = 0.299), teachers with a bachelor’s or master’s degree had higher median burnout, while those with a doctorate had the lowest (see Figure 6, Table 6).
- Preschool grade. Although not statistically significant (*p* = 0.662), teachers of first grade reported the highest median burnout, followed by third grade, while those teaching second grade had the lowest (see Figure 7, Table 7).
- Elementary grade. A significant difference was observed among elementary grades taught (*p* = 0.004; see Figure 8, Table 8). Burnout was highest among first grade teachers, followed by second and fifth grades, while the lowest burnout was found in third and sixth grades.

**Table 2:** Mann–Whitney *U* test for burnout level (urban vs. rural) (*n* = 637).

Mann–Whitney *U*	24,062.000
Wilcoxon W	29,522.000
*Z*	−2.130
Asymp. Sig. (2-tailed)	0.033
Total *N*	637

**Table 3:** Mann–Whitney *U* test comparing elementary vs. preschool teachers (*n* = 637).

Mann–Whitney *U*	48,561.500
Wilcoxon W	90,756.500
*Z*	−0.759
Asymp. Sig. (2-tailed)	0.448
Total *N*	637

**Table 4:** Mann–Whitney *U* test by gender (*n* = 637).

Mann–Whitney *U*	23,536.500
Wilcoxon W	28,096.500
*Z*	−1.336
Asymp. Sig. (2-tailed)	0.182
Total *N*	637

**Table 5:** Kruskal–Wallis test for marital status (*n* = 637).

Test statistic	2.968
Degrees of freedom	3
Asymptotic Sig. (2-sided test)	0.397
Total *N*	637

**Table 6:** Kruskal–Wallis test for education level (*n* = 637).

Test statistic	3.676
Degrees of freedom	3
Asymptotic Sig. (2-sided test)	0.299
Total *N*	637

**Table 7:** Kruskal–Wallis test for preschool grade taught (*n* = 290).

Test statistic	0.825
Degrees of freedom	2
Asymptotic Sig. (2-sided test)	0.662
Total *N*	290

**Table 8:** Kruskal–Wallis test for elementary grade taught (*n* = 347).

Median	1.000
Test statistic	17.048
Degrees of freedom	5
Asymptotic Sig. (2-sided test)	0.004
Total *N*	347

## Discussion

The primary aim of this study was to assess the comparative impact of both personal factors (such as age, years of teaching experience, marital status, and gender) and occupational factors (including the teacher’s academic degree, the educational level they work in, the specific grade they teach, the city of employment, and their perception of the school principal) on teacher burnout. The analysis utilizing decision tree methodology revealed that the variable “support, guidance, and positive attitude from the school principal” exerted the most significant influence on teacher burnout levels. Specifically, our findings unveiled a distinct pattern: teachers reported lower levels of burnout when they perceived frequent positive interactions with their principals, occurring several times a week or even daily.

Conversely, higher levels of burnout were associated with less frequent positive perceptions, categorized as never, rarely, or occasionally throughout the year or month. This aligns with prior research by Tornuk and Gunes (2020), which underscored the crucial role of perceived support from superiors, particularly school principals, in mitigating teacher burnout. Their study highlighted that a lack of support from principals was strongly correlated with elevated levels of burnout among teachers.

Additional evidence supports this pattern. Jentsch et al. (2023) found that teachers who rated the school principal’s leadership as high-quality were less likely to experience work-related stress and more likely to receive social support and regular feedback from colleagues. These positive conditions enhanced job-related self-efficacy and reduced the likelihood of burnout.

In the Mexican context, principals’ responsibilities extend beyond leadership to managing budgets and resources (Ortega-Muñoz et al., 2025). They ensure the availability of basic services, adequate infrastructure, and full staffing, including specialized personnel such as psychologists, sports instructors, and language teachers (Cóndor Surichaqui et al., 2024). Moreover, principals act as mediators between parents and teachers, preventing harassment from families and enforcing parental responsibilities by reporting cases of negligence to social services (Reyes, 2023).

This broader managerial role suggests that principals can influence nearly all dimensions of teacher burnout. By securing resources and shielding teachers from external pressures, they directly shape organizational climate and working conditions. Thus, effective principal leadership in Mexico represents not only pedagogical guidance but also systemic protection for teachers’ professional well-being.

The next most influential factors impacting teacher burnout levels include age and years of experience, both exhibiting an inverse relationship with burnout. These findings resonate with Teles et al. (2020), who observed that teachers leverage maturity and confidence to enhance coping resources. Similarly, Rasheed-Karim (2020) noted that older individuals tend to prioritize well-being and regulate emotions more effectively than younger colleagues.

The perception of the school principal being excessively demanding was particularly pronounced among teachers exhibiting the highest levels of burnout. Jones (2023) revealed that excessive expectations from principals increased stress, while supportive leadership promoted collaboration and trust. Teachers in such negative environments often expressed intentions to leave the profession, showing the severity of this factor.

Another statistically significant factor was the city where the teacher works. Burnout levels were higher in urban areas compared to rural ones, aligning with Dagar and Mathur’s (2016) findings in India. Similarly, Camacho and Parham (2019) and Saltijeral Méndez and Ramos Lira (2015) identified urban challenges such as aggression, lack of motivation, and disruptive behavior as major stressors.

In the present study, teachers responsible for teaching first and second-grade elementary students showed significantly higher burnout than other grade levels. This aligns with Skaalvik and Skaalvik (2017), who explained that younger students require more discipline management and time investment. These demands increase pressure and elevate burnout risk among teachers in the early grades.

The factors that showed no significant difference included gender, marital status, the level of education taught, the teacher’s highest level of education, and the grade of preschool taught. This outcome contrasts with Juárez García et al.’s (2020) findings but is consistent with Yilmaz et al. (2015). These results suggest that contextual and relational factors may outweigh demographic variables in predicting burnout.

The secondary objective was to quantify the overall level of teacher burnout and its dimensions using the SBI framework (Gil-Monte, 2005). Out of the 23.4% of teachers with high and critical burnout, 14.29% corresponded to profile 1, characterized by fatigue and indolence without severe guilt. In contrast, 9.11% corresponded to profile 2, defined by guilt, depressive symptoms, and risk of absenteeism.

This distribution aligns closely with Villaverde Echavarría et al. (2023), who reported 23.2% of teachers with critical burnout in a comparable sample. Profile 2 cases are particularly concerning, as they are associated with anxiety, depression, and long-term professional disengagement. Such findings highlight the urgent need for preventive and remedial interventions in school systems.

Overall, the study revealed a moderate level of burnout across all dimensions, consistent with Villaverde Echavarría et al. (2023) but higher than Castro et al. (2008). Rivera et al. (2021) and Garcia and Weiss (2019) attribute recent increases to worsening working conditions, including constant educational reforms, temporary contracts, absenteeism, and disruptive behaviors. These systemic stressors appear to compound the individual and organizational factors identified in this research.

## Conclusion

The findings of this study highlight the need to advance toward more differentiated and context-sensitive educational policies. First, it is recommended to implement specific strategies for urban schools, where levels of violence, disruptive behaviors, and family demands represent an additional risk factor for teacher well-being. It is also essential to provide specialized support in the first and second grades of primary school, drawing on the collaboration of teacher-training students, given that these levels entail a considerably higher burden of classroom management and discipline.

With regard to the selection of school principals, it is crucial to establish a clear professional profile that combines proven teaching experience with training in educational management or school leadership. The selection process should include competency-based evaluations (case studies, structured interviews) and be overseen by a plural committee that incorporates not only authorities but also experienced teachers and supervisors. Emphasis should be placed on pedagogical and human leadership, prioritizing the ability of principals to support and motivate their teaching staff.

Finally, regarding supervision and continuous professional development, it is suggested to implement periodic formative evaluations focused on qualitative observations and school community perceptions, along with constructive feedback aimed at professional growth. This should be complemented by mentorship and peer support among principals, mandatory continuous training in leadership and conflict management, and the establishment of individual improvement plans. Additionally, it is recommended to formalize mediation protocols that protect teachers from parental pressure or harassment, ensuring the active involvement of principals and social services. Taken together, these recommendations provide a realistic and feasible framework for preventing teacher burnout and strengthening the school climate from both an organizational and policy perspective.

## Data Availability

The datasets generated and analyzed in this study are not available due to ethical and privacy restrictions related to sensitive human-participant data. Further inquiries can be directed to the corresponding author.
